# Manganese Exposure Aggravates β-Amyloid Pathology by Microglial Activation

**DOI:** 10.3389/fnagi.2020.556008

**Published:** 2020-11-10

**Authors:** Geng Lin, Xinlu Li, Xiaofeng Cheng, Ning Zhao, Wei Zheng

**Affiliations:** ^1^Department of Histology and Embryology, China Medical University, Shenyang, China; ^2^Shengjing Hospital of China Medical University, Shenyang, China

**Keywords:** Alzheimer’s disease, β-amyloid, microglia, manganese, neuroinflammation, cytokines

## Abstract

Human epidemiological evidence and animal experimental data suggest that chronic manganese (Mn) exposure increases the risk of Alzheimer’s disease (AD) and amyloid plaques, a hallmark of AD brain pathology, but the underlying mechanisms were not fully understood. Using the transgenic APP/PS1/Tau triple transgenic AD (3×Tg-AD) mouse model and mouse-derived microglia and neuroblastoma cell lines, we found that chronic 5-month Mn treatment increased beta amyloid peptide (Aβ) expression and Aβ plaques in the cerebral cortex and hippocampus in these 3×Tg-AD mice. Furthermore, we found that the β- and γ-secretase cleavage activities were markedly increased, while α-secretase cleavage activity was reduced in the brain of Mn-treated AD mice; these effects increase Aβ production and thus are amyloidogenic. Equally important, Mn treatment alone did not alter β-secretase 1 (BACE1) gene expression or Aβ production in amyloidogenic mutant amyloid precursor protein (APP) gene hAPPsw-transfected N2a cells (APPsw-N2a), but in APPsw-N2a cells either co-cultured with microglia or cultured with microglia-conditioned media, Mn exposure increased BACE1 expression and amyloidogenesis. We further determined that Mn exposure promoted the activation of microglia both in 3×Tg-AD mouse brains and in cultured microglia cells, and increased the secretion of the inflammatory cytokines interleukin-1β (IL-1β) and tumor necrosis factor-α (TNF-α). Taken together, these results suggest that Mn may increase the release of IL-1β and TNF-α from microglia that in turn stimulates the expression of BACE1 gene and protein and consequently Aβ production; this novel molecular mechanism not only advances our understanding about the amyloidogenic effect of chronic Mn exposure reported for special human populations but also indicates Mn dyshomeostasis as a potential contributor to AD pathogenesis.

## Introduction

Alzheimer’s disease (AD) is the most common dementia-causing neurodegenerative disease that inflicts 5.8 million senior people in the United States alone and 40 million senior people worldwide, and the patient population is increasing as the life expectancy increases (Alzheimer’s Association, 2020). Thus, understanding the causes and risk factors for AD is critically important for the global fight against this disease. While old age is a key risk factor and abnormal Aβ and tau are key molecules causing AD neurodegeneration (Selkoe and Hardy, 2016; Forner et al., 2017; Walsh and Selkoe, 2020), other factors may also contribute to AD pathogenesis. One such contributing factor is a dyshomeostasis of essential biometals (e.g., iron, zinc, copper, and manganese; Jucker and Walker, 2013; Ciechanover and Kwon, 2015; Adlard and Bush, 2018; Soto and Pritzkow, 2018; Lermyte et al., 2019). Previous studies suggested that these biometals may disrupt and retard the metabolism, and facilitate the aggregation, of β-amyloid (Aβ) peptide and tau protein (Ayton et al., 2013; Guo et al., 2013; Tong et al., 2014; Ward et al., 2014; Li et al., 2017; Takeda et al., 2017; Cheignon et al., 2018), but firm conclusions and mechanisms about the functional roles of these essential biometals in AD pathogenesis are not established.

Manganese (Mn) is an essential trace metal in the human body and has important biochemical and physiological functions as it is a cofactor for several important enzymes, such as glutamine synthetase, pyruvate carboxylase, arginase, and Mn superoxide dismutase (Chen et al., 2015). However, elevated Mn levels can impair the function and structure of the brain, as demonstrated by the consequences from the sustained high Mn level after the inactivation of the transmembrane transporter for Mn (Bowler et al., 2007; Ellingsen et al., 2008; Guarneros et al., 2013; Jenkitkasemwong et al., 2018; Mukhopadhyay, 2018). Mn is present in the air, soil, and waterways and can enter the human body *via* breathing, food, and water (Horning et al., 2015; Peres et al., 2016). The healthy brain is capable of efficiently regulating Mn homeostasis under physiological conditions (Chen et al., 2015; Jenkitkasemwong et al., 2018; Mukhopadhyay, 2018; Taylor et al., 2019); however, Mn overexposure, due to excessive Mn in the natural environment and occupational environments such as mining and welding, can lead to its increased accumulation in the central nervous system. Mn accumulation in the basal ganglia and its associated neurotoxicity can result in manganism characterized by parkinsonian motor deficits (Yamada et al., 1986; Racette, 2014). Studies have also found cognitive deficits in human populations living near refineries with high Mn levels (Guarneros et al., 2013), and there is dose–effect relationships between Mn exposure and cognitive decline (Bowler et al., 2007; Ellingsen et al., 2008).

Furthermore, chronic Mn exposure was reported to increase the expression of amyloid-beta precursor-like protein 1 (APLP1) gene and protein, and the formation of diffuse Aβ plaques in the frontal cortex of nonhuman primates (Guilarte et al., 2008; Guilarte, 2010). Additionally, Mn was shown to be at significantly higher levels in the brain of AD patients compared to healthy subjects, while the highest level was detected in the parietal cortex where AD pathology is severe (Srivastava and Jain, 2002; Tong et al., 2014; Cheignon et al., 2018), and there was a significant increase in Aβ peptides correlated with Mn both in the plasma of AD patients and in the brain of AD mouse models (Tong et al., 2014). Together, these literature data suggest that Mn overload may be a potential risk for AD and is involved in the pathogenesis of AD and cognitive dysfunction, but the underlying mechanisms for Mn to increase Aβ pathology are not fully established.

To further determine Mn’s potential enhancement of AD pathology and the underlying molecular mechanisms, our present study investigates the effects of chronic Mn exposure on amyloid plaque formation in mutant APP/PS1/Tau triple transgenic AD mice and cultured cells expressing mutant amyloidogenic amyloid precursor protein (APP) and the involvement of brain innate immune cells, microglia; this combined approach enables us to study the potential Mn effects in intact brain tissues—conferring more pathophysiological relevance, and also in isolated cultured cells—allowing more detailed cellular and molecular analyses.

## Materials and Methods

### Transgenic Mice and Mn Treatment Regimen

Breeders of the APP/PS1/Tau triple transgenic AD (3×Tg-AD) mouse model, originally created by Oddo et al. (2003), was obtained from the Jackson Laboratory (Stock No.: 34830, Bar Harbor, ME, USA). All mice were kept in a controlled environment (22–25°C room temperature, 12 h light/dark cycle, and 40–60% relative humidity) with free access to water and food. The Laboratory Animal Ethics Committee of China Medical University approved all experimental procedures. Only male mice were used because cyclic hormonal changes in female mice may affect the production, metabolism, and accumulation of APP and Aβ and consequently confound data interpretation. Future studies need to investigate the potential sex differences in Mn effects on amyloidogenesis.

Because in 3×Tg-AD mice, amyloidogenesis and Aβ pathology progress through 2–26 months (Oddo et al., 2003; Mastrangelo and Bowers, 2008), we chose 8 months of age as the starting point. Thus, 8-month-old male 3×Tg-AD mice were randomly divided into two groups: mice of Mn group were provided with Mn-containing drinking water (108 mg MnCl_2_•4H_2_O dissolved in 300 ml of distilled drinking water; MnCl_2_•4H_2_O, 99% purity was purchased from Sigma–Aldrich, St. Louis, MO, USA); mice of the Control group were provided with distilled drinking water. Mice were monitored daily, and their body weight was measured and recorded weekly. At the end of the 5-month-treatment, whole blood samples were collected directly from the heart of the mice deeply anesthetized with intraperitoneally injected 4% chloral hydrate at 0.1 ml/10 g of body weight, and the serum was prepared; this blood collection method is well established for collecting the maximal amount of blood with minimal contamination. The Mn concentrations in sera and brains were determined using mass spectrometry as detailed below.

### Cell Culture

Mouse N2a neuroblastoma cells stably transfected with human Swedish mutant APP (abbreviated as APPsw-N2a cells for convenience) were provided by Dr. Huaxi Xu of Sanford Burnham Prebys Medical Discovery Institute, USA. Mouse BV2 microglia cells were provided by Dr. Yuhua Chen of China Medical University. The APPsw-N2a cells were maintained in 6-cm tissue culture dishes in normal DMEM medium supplemented with 10% fetal bovine serum (Gibco, Carlsbad, CA, USA) and selected by 200 μg/ml of G418, following the methods in the literature (Guo et al., 2017). For studying the impact of Mn and inflammatory factors on the amyloidogenesis of APPsw-N2a cells, these cells were treated with 0, 100, and 500 μM MnCl_2_ for 24 h, then the cells were collected for analyses.

### Transwell Coculture APPsw-N2a Cells With BV2 Microglia Cells

To study the effects of microglia-secreted factors on APP processing, BV2 microglia were seeded onto permeable Transwell cell culture inserts (BD Biosciences, Franklin Lakes, NJ, USA) at 1 × 10^5^ cells/well, APPsw-N2a cells were at 1 × 10^6^ cells/well, and they were allowed to adhere for 48 h. These Transwell inserts are permeable to cell/microglia-secreted factors, while the cultured cells in the two compartments do not mix and can be collected separately. Cell culture medium was changed to fresh serum-free Mn-free DMEM 24 h prior to treatment. BV2 inserts were placed into six-well culture plates seeded with APPsw-N2a, and both were treated with either 0 or 100 μM Mn for 24 h. Then the APPsw-N2a cells were collected for Western blot analysis.

### Microglia-Conditioned Cell Culture Media Preparation

BV2 microglia cells were seeded onto six-well tissue culture plates at 1 × 10^5^ cells/well and treated with 0 and 100 μM MnCl_2_ for 24 h. Then the culture supernatants as microglia-conditioned media (MCM) were aspirated and centrifuged at 800× *g* for 10 min to remove detached cells. MCM was fed to APPsw-N2a cells cultured in 60-mm tissue culture dishes at 1 × 10^6^ cells/well with no additional Mn added for another 24 h. The 1:10 ratio of microglia to neurons was utilized based on previous reports (Pelvig et al., 2008; Correa et al., 2013; Herculano-Houzel, 2014).

### Measurement of Brain Mn Levels

**Figure 1:**
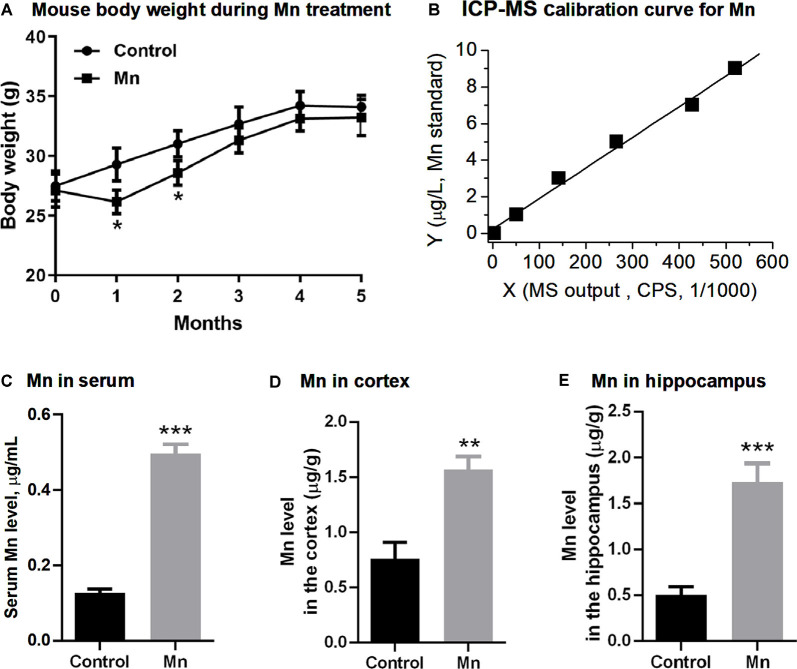
Manganese (Mn) treatment *via* Mn-containing drinking water increased Mn concentrations in sera and brains in 3×Tg-AD mice. **(A)** The body weights of the mice were decreased in the Mn group in the first and second month, and then regained their weights in the following 3 months. *N* = 10 male 3×Tg-AD mice for each group. *p < 0.05, repeated measure (RM) ANOVA. Additional male 3×Tg-AD mice were similarly treated, but their body weight was only measured before euthanization and was normal. **(B)** Inductively coupled plasma mass spectrometer (ICP-MS) calibration curve using Mn standards. **(C,D)** ICP-MS measurements of the Mn level in the sera **(C)**, the cerebral cortex **(D)**, and the hippocampus **(E)** in control 3×Tg-AD mice and 3 Mn-treated 3×Tg-AD mice. **p < 0.01, ***p < 0.001, *t*-test.

We used an inductively coupled plasma mass spectrometer (ICP-MS; model 7500a, Agilent Technologies Inc., CA, USA) to measure the Mn level in brain tissues. The cortical and hippocampal tissue samples (20 mg for each wet tissue sample) were digested by adding 500 μl of nitric acid; then the completely digested samples were diluted 20 times. Following the user manual, the instrument was operated at a radiofrequency power of 1,420 W, the argon carrier gas flow rate was 1.05 l/min, and the argon plasma gas flow rate was 15 l/min. Using the peak area mode, the data acquisition time was 2 s, and three measurements were repeated. ICP-MS measurements of Mn in blood and brain tissue samples were converted to concentrations based on the calibration curve and its linear fit equation and sample dilution (Figure 1B). The reliability of our measurements is confirmed by the fact that our baseline Mn levels were similar to the blood and brain Mn levels reported in the literature (Garcia et al., 2006; Moldovan et al., 2013; Jenkitkasemwong et al., 2018).

### Cytotoxicity Assay

Mn cytotoxicity was assessed by cell counting kit-8 (CCK-8; Cat.# B34304, Bimake, Houston, TX, USA) following the manufacturer’s instructions. Wild-type N2a cells were seeded in a 96-well plate at a density of 5 × 10^4^ cells/well in 100 μl of culture medium and were cultured for 24 h. Then the culture medium was changed to serum-free medium containing MnCl_2_ at 50, 100, 200, 500, 800, and 1,000 μM. The cells were cultured in a CO_2_ incubator at 37°C for another 24 h. Ten microliters of CCK8 was added to the culture for 2 h at 37°C. The optical density (OD) was measured at 450 nm with a microplate reader (Infinite 200 Pro, TECAN, Grödlg, Austria). Cell viability was calculated using the method described in the CCK-8 kit. Following the method of Daoust et al. (2014), a linear fit to the cell viability data points estimated the 50% lethal concentration (LC_50_) to be 687.0 μM MnCl_2_ for N2a cells under our experimental condition, roughly similar to the reported LC_50_ of 863 μM MnCl_2_ for N2a cells (Daoust et al., 2014); at 100 μM, Mn did not induce any detectable cytotoxicity. Thus, we used 100 μM MnCl_2_ in most of our experiments on cultured cells.

### Immunofluorescence

For double-immunofluorescent staining and confocal microscopic analysis, frozen sections were preincubated with normal sheep serum for 1 h and then incubated overnight at 4°C in a mixture of primary antibodies for mouse anti-Aβ (1:1,000) and rabbit anti-Iba1 (1:500). After several rinses with PBS, the sections were incubated for 2 h at room temperature with a mixture of FITC (green) and TRITC (red)-conjugated secondary antibodies (1:400; ZSGB-BIO, Los Altos, CA, USA) followed by mounting and coverslipping. The images were observed and captured using a confocal laser scanning microscope (Nikon C1, Tokyo, Japan). The fluorescence signal was absent when the primary antibody was omitted, indicating the specificity of the antibody. The primary antibodies used in this study, their sources, and dilutions are listed in Table 1.

**Table 1:** Primary antibodies.

Antibody name	Dilution	Host	Source/vendor	Cat no.	Application
ADAM10	1:1,000	Rabbit	Millipore	AB 19026	Western blotting
APP	1:1,000	Mouse	Cell Signaling	NAB228	Western blotting
BACE (10E5)	1:1,000	Rabbit	Cell Signaling	5606S	Western blotting
Iba1	1:500	Rabbit	Wako	019-19741	IHC
IL-1β	1:1,000	Rabbit	Proteintech	16806-1-AP	Western blotting
Presenilin-1	1:1,000	Mouse	Abcam	ab15456	Western blotting
TNF-α	1:1,000	Rabbit	Proteintech	17590-1-AP	Western blotting
β-actin	1:10,000	Mouse	Sigma–Aldrich	A1978	Western blotting
β-amyloid	1:1,000	Mouse	Sigma–Aldrich	A5213	IHC
β-amyloid (D54D2)	1:1,000	Rabbit	Cell Signaling	8243s	Western blotting

### Western Blot

After addition of the protease inhibitor cocktail, the samples were subjected to immunoblot analysis. The protein concentration was measured with a bicinchoninic acid (BCA) protein assay kit (Cat.# P0012, Beyotime, Shanghai, China). The total protein lysate (30 μg) was separated *via* 10% sodium dodecyl sulfate (SDS) polyacrylamide gels and transferred on polyvinylidene fluoride (PVDF) sheets (Millipore, Burlington, MA, USA). The membranes were blocked by 5% (w/v) nonfat milk. The following antibodies were used: rabbit anti-β-amyloid (D54D2; 1:1,000, Cat.# 8243S; Cell Signaling, MA, USA), mouse anti-APP (1:1,000, Cat.# NAB228, Cell Signaling, MA, USA), mouse anti-presenilin 1 (1:1,000, Cat.# Ab15456; Abcam, MA, USA), rabbit anti-ADAM10 (1:1,000, Cat.# AB19026; Millipore, Burlington, MA, USA), rabbit anti-BACE (D10E5; 1:1,000, Cat.# 5606S; Cell Signaling, MA, USA), rabbit anti-TNF-α (1:1,000, Cat.# 17,590-1-AP, Proteintech, USA), rabbit anti-IL-1β (1:1,000, Cat.# 16,806-1-AP, Proteintech, Chicago, IL, USA), and mouse anti-β-actin (1:10,000, Cat.# A1978, Sigma–Aldrich, St. Louis, MO, USA). After washing three times with Tris-buffered saline containing Tween-20 for 5 min each, the membranes were incubated with appropriate HRP-conjugated secondary antibodies (1:5,000; Cell Signaling, MA, USA) for 1 h at room temperature. Membranes were washed for 5 min three times with TBST after incubation with each antibody. Finally, immunological complexes were visualized by an enhanced chemiluminescence reagent (Cat.# 34080, Pierce, Thermo Fisher Scientific, Waltham, MA, USA) using a ChemiDoc XRS system (Tanon-5200, BioTannon Company, Shanghai, China). The immunoreactive bands were quantified using Image-pro Plus 6.0 analysis software. The antibodies used for Western blot are listed in Table 1.

### Enzyme-Linked Immunosorbent Assay

The production of soluble Aβ1–40 (RayBiotech, Norcross, GA, USA), Aβ1–42 (Cusabio Biotech LLC, Houston, TX, USA), TNF-α (eBioscience Inc., San Diego, CA, USA) and IL-1β (eBioscience Inc., San Diego, CA, USA) in the culture supernatants was measured by enzyme-linked immunosorbent assay (ELISA) following the manufacturer’s instructions.

### Semiquantitative RT-PCR of mRNAs

Total RNA was isolated using Trizol reagent (Invitrogen, Cat.# 15596018) according to the product’s user instructions. RNA purity and quality was monitored by 260/280 nm OD ratios. The cDNA was synthesized from 2 μg total RNA using the GoScript Reverse Transcription System (Cat.# A5003, Promega, Madison, WI, USA) on the Bio-Rad CFX PCR System. The primer sequences are listed in Table 2. The housekeeping gene GAPDH was used as an internal control. The relative mRNA expression level was calculated by Bio-Rad CFX software using the ΔΔCt method (Heid et al., 1996; Xue et al., 2015).

**Table 2:** RT-PCR primers (for mouse genes).

ADAM10 forward: GCACCTGTGCCAGCTCTGAT ADAAM10 reverse: TCCGACCATTGAACTGCTTGT
APP forward: TGATCTACGAGCGCATGAAC APP reverse: AGA AGGCATGAGAGCATCGT
BACE1 forward: GGATTATGGTGGCCTGAGCA BACE1 reverse: CGTGTCCACCAGGATGTTGA
GAPDH forward: TTCACCACCATGGAGAAGGC GAPDH reverse: GGCATGGACTGTGGTCATGA
PS1 forward: GCCCCAGAGTAACTCAAGACA PS1 reverse: CCGGGTATAGAAGCTGACTGA

### Statistical Analyses

The results are represented as mean ± SEM. All statistical analysis was performed using GraphPad Prism 6.0 software (GraphPad Software, Inc., San Diego, CA, USA). Student’s *t*-test was used for comparison of two groups. One-way ANOVA followed by a Tukey–Kramer multiple comparison *post hoc* test was utilized for comparison of three or more groups. Two-way ANOVA followed by Tukey–Kramer multiple comparison *post hoc* tests was adopted for comparison of three or more groups and two different variables in analyzing N2a cell responses in coculture and conditioned media experiments. Independent variables for two-way ANOVA were defined as treatment (normal drinking water vs. Mn-containing drinking water) and microglial presence (Mn alone vs. microglia + Mn). Repeated measure ANOVA (RM ANOVA) was used for comparing the body weight of the two groups of mice at different time points. A value of *p* < 0.05 was considered as statistically significant.

## Results

### Chronic Oral Mn Administration Increases Blood and Brain Mn Concentrations

We first measured and analyzed the body weight of 3× Tg-AD mice receiving Mn in their drinking water. The body weight of the mice in Mn group was lower than that of the control group at the end of the first month and the second month (*p* < 0.05, RM ANOVA; Figure 1A); the body weight of the two groups was not significantly different during the next 3 months (*p* > 0.05, RM ANOVA; Figure 1A). Because a metallic taste of Mn-containing water is well documented (Thomsen et al., 2004; Saha et al., 2015), we speculate that the lower body weight in the first 2 months might be because the mice did not like the Mn-containing, metallic-tasting Mn-containing drinking water and drank and ate less—although we did not monitor the water and food intake; and after the first 2 months, these mice became used to the Mn-containing drinking water, drank and ate normally, and regained their body weight. Indeed, a similar drinking water Mn-induced body weight loss was reported in normal mice in a previous study (Krishna et al., 2014) that showed that drinking water Mn initially reduced water intake and body weight, but these effects disappeared after 7 weeks.

Our mouse experiments below were conducted at the end of the 5-month Mn treatment when the 3×Tg-AD mice in the Mn treatment group had normal body weight, and their general peripheral health was likely comparable to that of the control (non-Mn treated) 3×Tg-AD mice.

We next used ICP-MS to determine Mn levels in sera and brains of 3×Tg-AD mice (Figure 1B). The serum Mn level in the Mn-treated group of 3×Tg-AD mice was 0.496 ± 0.015 μg/ml (*n* = 3 mice), compared with the control group’s 0.128 ± 0.006 μg/ml (*n* = 3 mice, *p* < 0.001, *t*-test; Figure 1C). The Mn level in the cerebral cortex (parietal area) was 1.57 ± 0.07 μg/g wet tissue in the Mn-treated 3×Tg-AD mice (*n* = 3) and 0.75 ± 0.09 μg/g wet tissue in the control group (*n* = 3); the hippocampus Mn level was 1.74 ± 0.12 μg/g wet tissue in the Mn-treated 3×Tg-AD mice (*n* = 3) and 0.51 ± 0.05 μg/g wet tissue in the control group (*n* = 3). Statistical analysis showed that long-term Mn administration *via* drinking water significantly increased the Mn concentrations both in the cortex and in the hippocampus (*n* = 3, *p* < 0.01, *t*-test; Figures 1D,E). Also, our baseline blood and brain Mn levels were similar to the reported values (Garcia et al., 2006; Moldovan et al., 2013; Jenkitkasemwong et al., 2018), indicating the reliability of our Mn measurements.

### Effects of Chronic Mn Treatment on APP and Aβ Expression in 3×Tg-AD Mouse Brains

**Figure 2:**
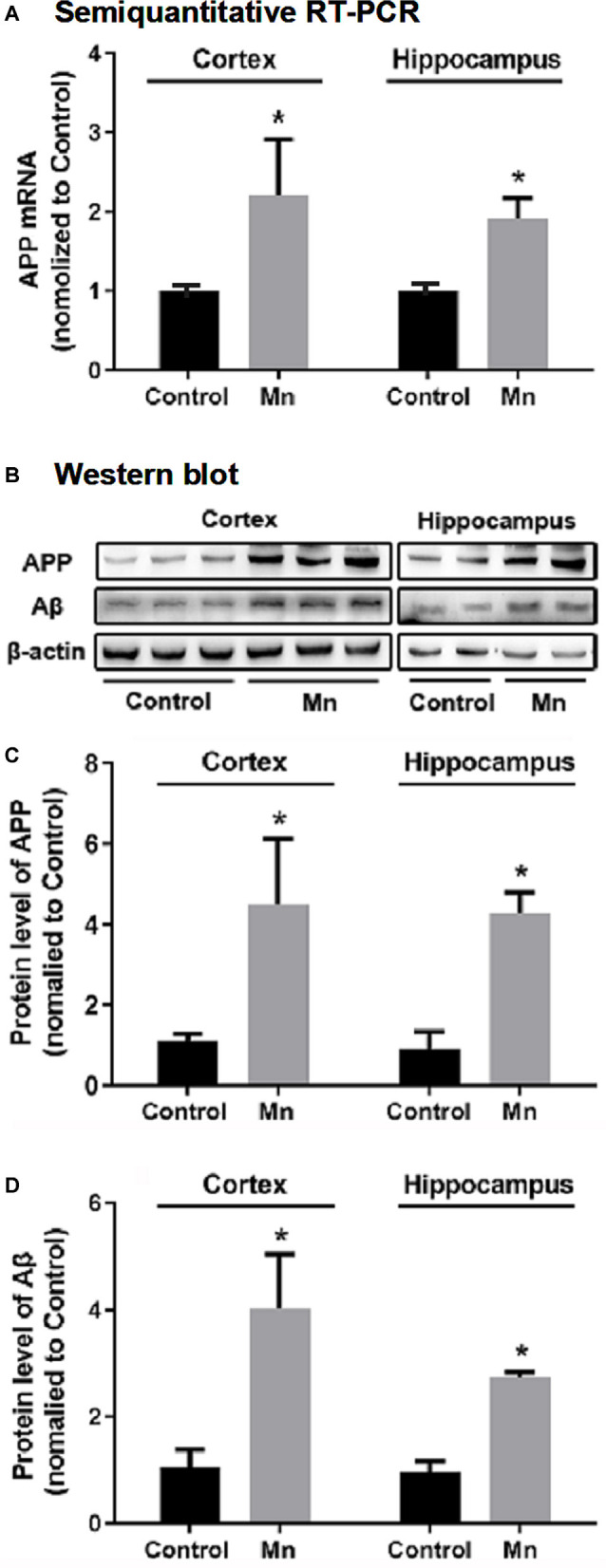
Chronic Mn treatment increased β-amyloid (Aβ) production in 3×Tg-AD mouse brains. **(A)** APP mRNA level in 3×Tg-AD mouse brains, determined by semiquantitative RT-PCR with GAPDH mRNA serving as the internal control, was increased in the cerebral cortex and hippocampus of Mn-treated 3×Tg-AD mice. **(B–D)** The protein expression of APP and Aβ in 3×Tg-AD mouse brain, determined by Western blot, was increased in the cortex and hippocampus of Mn-treated 3×Tg-Ad mice. β-actin was used as the internal control. *n* = 5 mice for each group. *p < 0.05, compared with the control group, *t*-test.

To test whether high brain Mn affects APP expression, the levels of APP mRNA and full-length APP protein were measured by semiquantitative RT-PCR and Western blot, respectively. As shown in Figure 2A, APP mRNA levels were increased both in the cerebral cortex (to 221.50 ± 40.20%, *n* = 5) and in the hippocampus (to 191.20 ± 13.16%, *n* = 5) of the Mn group compared with the control (*p* < 0.05, *n* = 5, *t*-test). Western blot analysis showed that Mn treatment significantly increased APP protein level to 447.58 ± 95.45% in the cerebral cortex (*n* = 5) and to 439.10 ± 50.79% in the hippocampus (*n* = 5), compared with control group (*n* = 5, *p* < 0.05, *t*-test; Figures 2B,C).

To determine whether long-term oral Mn intake altered Aβ levels in the brain of 3×Tg-AD mice, an Aβ antibody (Cat.# 8243s, Cell Signaling Technology, see Table 1) was used in Western blot for detecting and quantifying Aβ fragments (Figure 2B). Statistical analysis showed that the chronic Mn treatment *via* drinking water significantly increased the Aβ level to 402.17 ± 106.92% in the cerebral cortex (*n* = 5) and to 278.27 ± 12.40% in the hippocampus (*n* = 5), compared with the control group (*n* = 5, *p* < 0.05, *t*-test; Figures 2B,D). Taking together, our data presented above indicate that high brain Mn increased both APP gene and protein expression and also Aβ production and accumulation. The question now is: how?

### Mn Effects on Amyloidogenic APP Processing in 3×Tg-AD Mice

**Figure 3:**
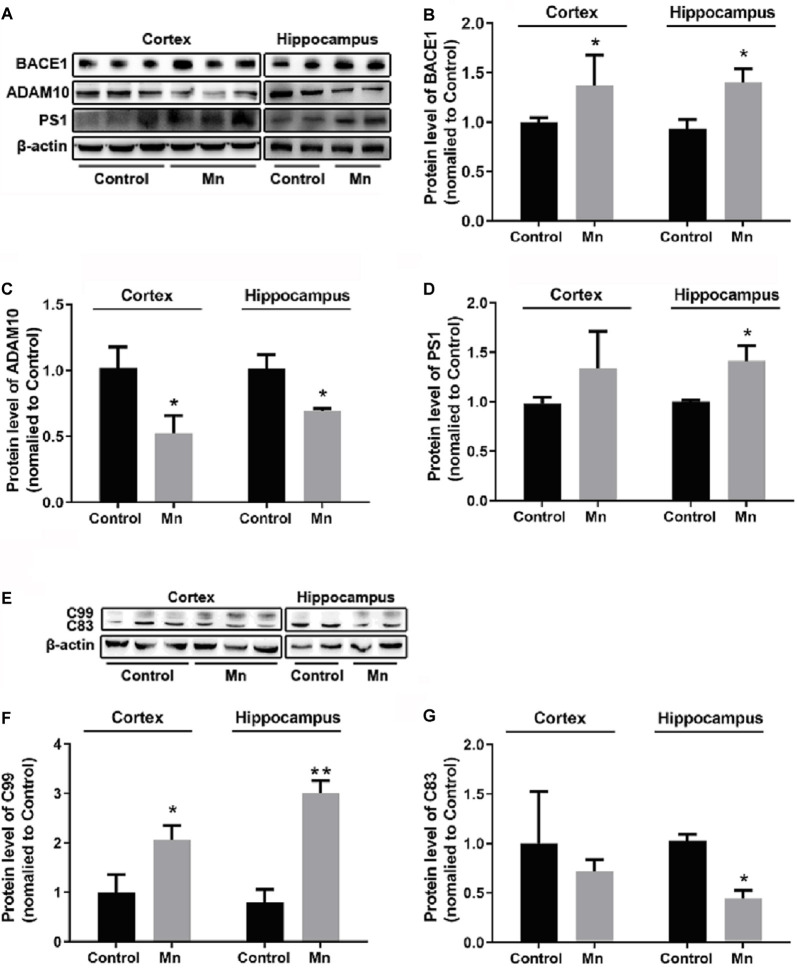
Mn exposure promoted amyloidogenic APP processing in 3×Tg-AD mouse brains. **(A–D)** The expression levels of BACE1, ADAM10, and γ-secretase PS1 in the brain of Alzheimer’s disease (AD) were examined by Western blot. The protein levels of BACE1 and PS1 were significantly increased **(B,D)**, whereas ADAM10 were markedly decreased **(C)** in the cortex and hippocampus of Mn-exposure mice. **(E–G)** Western blot analysis of the expression of intracellular APP cleavage fragments, including C99 and C83, in the brain of AD mice treated with or without Mn. Mn exposure significantly increased the levels of C99 both in the cortex and hippocampus, and reduced the levels of C83 in hippocampus. β-actin served as the internal control. *n* = 5 mice for each group, *p < 0.05, **p < 0.01, compared with the control group, *t*-test.

To examine how high Mn intake increased amyloidogenic APP processing, qRT-PCR and Western blot analyses were performed to detect and quantify the key enzymes in APP processing including BACE1 (β-secretase activity producing Aβ), ADAM10 (a key component of α-secretase activity), presenilin 1 (PS1, a key component of α-secretase activity producing Aβ), and APP cleavage fragments including C99 and C83, in the cortex and hippocampus of control and Mn-treated 3×Tg-AD mice. As shown in Figure 3, Mn treatment significantly increased protein levels of β-secretase 1 BACE1 in the cerebral cortex and hippocampus of 3×Tg-AD mice (*p* < 0.05, *t*-test; Figures 3A,B); in contrast, protein levels of ADAM10 in the cortex and hippocampus of 3×Tg-AD mice were decreased significantly after Mn treatment (*n* = 5, *p* < 0.05, *t*-test; Figures 3A,C). Furthermore, the levels of PS1 in Mn-treated 3×Tg-AD mouse hippocampus and cortex were significantly increased compared with the control group (*n* = 5, *p* < 0.05, *t*-test; Figures 3A,D). Then we examined the levels of β-secretase-generated C99 fragment and α-secretase-generated C83 fragment in 3×Tg-AD mouse brains. Mn treatment significantly increased the levels of C99 fragment (*n* = 5, *p* < 0.05, *t*-test; Figures 3E,F) and reduced the levels of C83 fragment (*p* < 0.05; Figures 3E,G). Taken together, these results indicate that the β- and γ-secretase cleavage activities were markedly increased, while α-secretase cleavage activity was reduced in the cerebral cortex and hippocampus of Mn-treated 3×Tg-AD mice, and these chronic Mn treatment-induced changes are amyloidogenic.

### Mn Effects on Amyloidosis in N2a Cells Transfected With hAPPsw Requires Coculture With Microglia

**Figure 4:**
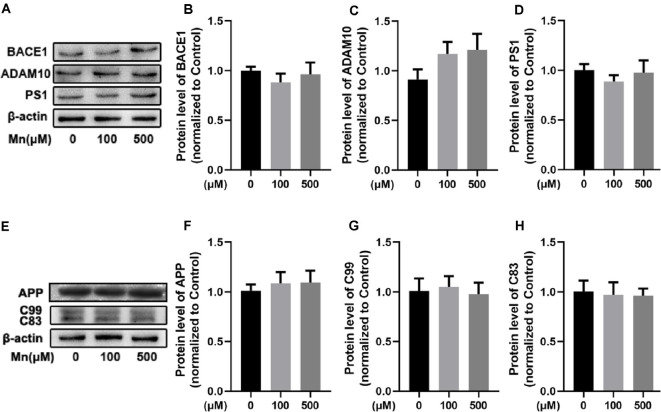
Mn-exposure did not influence amyloidogenic processing of APP in N2a cells transfected with APPsw. **(A–D)** Western blot analysis was carried out to examine the expression levels of APP cleavage enzymes, BACE1, ADAM10, and PS1, in APPsw-N2a cells. β-actin was used as an internal control. No significant change was detected in the protein levels of BACE1, ADAM10, and PS1 in 100 or 500 μM Mn-treated APPsw-N2a cells. **(E–H)** Western blot analysis was used to determine the protein levels of C99 and C83 in 100 or 500 μM Mn-treated APPsw-N2a cells. There were no significant changes in the C99 and C83 level in Mn-treatment cells compared with the controls. *n* = 5 mice for each group, *p* > 0.05, one-way ANOVA.

Next, we performed experiments in a mouse neuroblastoma N2a cell line stably transfected with amyloidogenic mutant APP hAPPsw gene (Guo et al., 2017) to further investigate the molecular mechanisms by which chronic Mn treatment affects APP processing for increased amyloidogenic Aβ production. Mn concentrations of 100 μM were used in most of our experiments, although 500 μM Mn was used for some experiments, based on the evaluation of cell viability assay and previous studies (LC50 ~800 μM: Daoust et al., 2014; Wang et al., 2017; Yin et al., 2018). Surprisingly, Mn exposure at these concentrations did not change the levels of APP cleavage enzymes (ADAM10, BACE1, and PS1) in APPsw-N2a cells (*n* = 5, *p* > 0.05, one-way ANOVA; Figures 4A–D); neither the level of APP protein nor its C-terminal fragment intermediates C99 and C83 were changed in APPsw-N2a cells (*n* = 5, *p* > 0.05, one-way ANOVA; Figures 4E–H). These results indicate that by itself, Mn was insufficient to affect Aβ production in APPsw-N2a cells.

**Figure 5:**
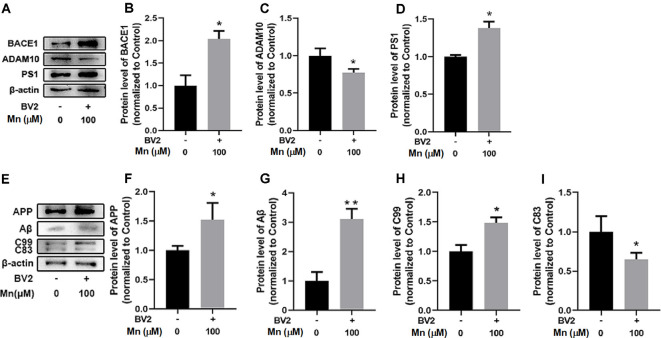
Mn enhances amyloidogenic APP processing in APPsw-N2a cells cocultured with microglia BV2 cells. **(A–D)** Western blot analyses of the protein levels of BACE1, ADAM10, and PS1 in APPsw-N2a cells. Example gel images are shown with β-actin as the internal control **(A)**. BACE1 level **(B)** and PS1 level **(D)** were increased, but ADAM10 level **(C)** was decreased, in 100 μM Mn-treated APPsw-N2a cells cocultured with BV2, compared with the control group. **(E–I)** Western blot analyses were carried out to examine the protein expression levels of APP, C99, C83, and Aβ in APPsw-N2a cells **(E)**. Mn treatment, 100 μM, significantly increased the protein levels of APP **(F)**, Aβ **(G)**, and C99 **(H)** in APPsw-N2a cells cocultured with BV2, compared with the control group; in contrast, 100 μM Mn treatment significantly decreased in the protein levels of C83 **(I)** in APPsw-N2a cells cocultured with BV2 compared with the control group. All data are means ± SE. *p < 0.05, **p < 0.01, two-way ANOVA, *n* = 5 for each group.

To test whether Mn and factors released from microglia can work together to affect Aβ production, we repeated the experiment in APPsw-N2a cells cocultured with mouse BV2 microglial cells. Under this condition, Mn treatment (100 μM for 24 h) significantly increased BACE1 by 103.84 ± 16.42% (*n* = 5) and PS1 by 37.75 ± 5.33% (*n* = 5; *p* < 0.05), and reduced ADAM10 by 22.77 ± 6.20% (*p* < 0.05, two-way ANOVA), respectively, in APPsw-N2a cells (Figures 5A–D); APP, C99, and Aβ were also increased by 51.89 ± 17.27% (*n* = 5), 48.21 ± 8.28% (*n* = 5) and 210.26 ± 27.14% (*n* = 5), respectively (*p* < 0.05), while the non-amyloidosis-processing product C83 was decreased by 35.40 ± 12.52% (*n* = 5; *p* < 0.05, two-way ANOVA; Figures 5E–I).

**Figure 6:**
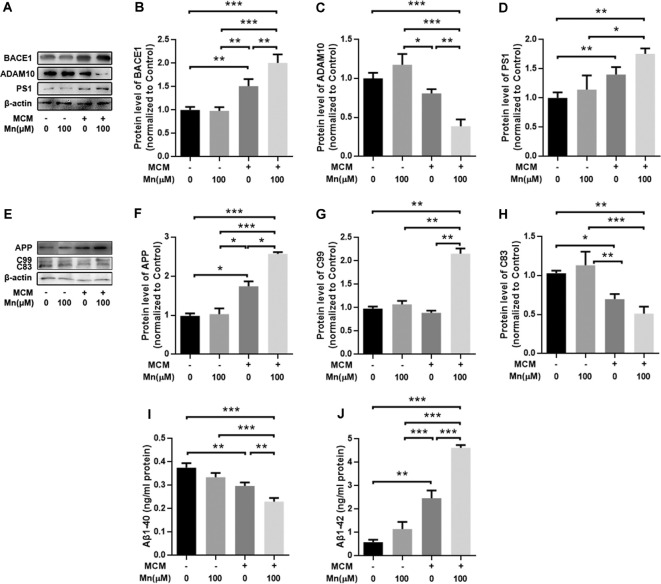
Amyloidogenic processing of APP was enhanced in APPsw-N2a cells cultured with microglia-conditioned media (MCM) from Mn-treated BV2 cells. **(A–D)** Western blot results on the expression levels of APP cleavage enzymes, BACE1, ADAM10, and PS1 in APPsw-N2a cells under different conditions. MCM, especially MCM from Mn-treated BV2 cells, significantly increased the levels of BACE1 **(B)** and PS1 **(D)**. MCM from Mn-treated BV2 cells also significantly decreased the level of ADAM10 **(C)** in APPsw-N2a cells. **(E–H)** Western blot results on the expression levels of APP, C99, and C83 in APPsw-N2a cells. β-actin was used as an internal control. APP and C99 levels were increased in APPsw-N2a cells cultured with MCM from Mn-treated BV2 cells compared with vehicle, Mn alone, MCM alone groups **(F,G)**. C83 levels were decreased in APPsw-N2a cells cultured with MCM from Mn-treated BV2 cells compared with the other three groups **(H)**. **(I,J)** Enzyme-linked immunosorbent assay (ELISA) data showing that Mn, MCM, especially MCM from Mn-treated BV2 cells increased Aβ1–42 secretion **(I)** and decreased Aβ1–40 production **(J)** in APPsw-N2a cells. *p < 0.05, **p < 0.01, ***p < 0.001, two-way ANOVA, *n* = 5 for each group.

To further determine how Mn and microglia work together to promote amyloidogenic APP processing in APPsw-N2a cells described above, we treated APPsw-N2a cells with microglia-conditioned media (MCM) from BV2 microglia with or without Mn treatment. Protein Western blot showed that treatment of APPsw-N2a cells with 100 μM Mn did not change the levels of APP, BACE1, ADAM10, PS1, Aβ1–42, and Aβ1–40 compared with the control (Figure 6). Exposure to MCM from normal microglia increased BACE1 (*n* = 5, *P* < 0.05, two-way ANOVA, Figures 6A,B) and PS1 (*n* = 5, *p* < 0.05, two-way ANOVA; Figures 6A,D), whereas a slight, but not significant, decrease in the level of ADAM10 (*p* > 0.05, two-way ANOVA; Figures 6A,C) compared with the control group. MCM treatment increased APP (*n* = 5, *p* < 0.05, two-way ANOVA; Figures 6E,F) and Aβ1–42 (*p* < 0.05, two-way ANOVA; Figure 6J), but reduced C83 (*n* = 5, *p* < 0.05; Figures 6E,H) and Aβ1–40 (*p* < 0.05, two-way ANOVA; Figure 6I). Additionally, treatment with MCM from 100 μM Mn-exposed microglia further increased BACE1 (*n* = 5, *p* < 0.01, two-way ANOVA; Figures 5A,B) and PS1 (*p* < 0.05, two-way ANOVA; Figures 6A,D) but decreased the level of ADAM10 (*p* < 0.05, two-way ANOVA; Figures 6A,C) in APPsw-N2a cells compared with both vehicle control and Mn-treatment alone. Furthermore, treatment with MCM from 100 μM Mn-exposed microglia also increased C99 (*p* < 0.05, two-way ANOVA; Figures 6E,G) and Aβ1–42 (*n* = 5, *p* < 0.05, two-way ANOVA; Figure 6J), but reduced C83 (*n* = 5, *p* < 0.05; Figure 6H) and Aβ1–40 (*n* = 5, *p* < 0.05, two-way ANOVA; Figure 6I) compared with the vehicle control, Mn-treatment alone, and MCM from normal microglia, respectively. Taken together, these results indicate that activation of microglia and their consequent release of inflammatory cytokines may be involved in the amyloidogenic effect of chronic Mn treatment.

### Mn Effects on Inflammatory Responses in 3×Tg-AD Mouse Brains and BV2 Microglial Cells

**Figure 7:**
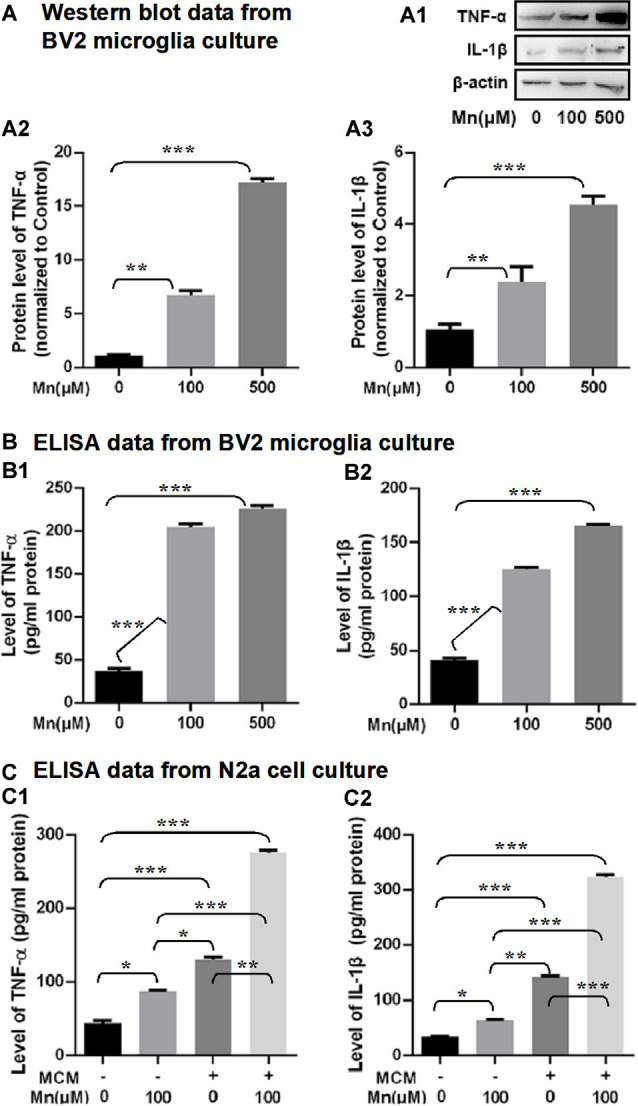
Effects of Mn exposure on the secretion of TNF-α and IL-1β in BV2 microglia and APPsw-N2a cells treated with a conditioned medium collected from BV2 cells in the absence or presence of 100 μM Mn for 24 h. **(A: A1–A3)** Western blots showing that 100 and 500 μM Mn treatment for 24 h each increased the protein levels of TNF-α and IL-1β in BV2 cells. β-actin served as internal control. **(B: B1,B2)** ELISA data showing that both 100 μM Mn and 500 μM Mn treatment for 24 h increased the secretion and release of TNF-α and IL-1β in BV2 cells. **(C: C1,C2)** ELISA data showing that the secretion of TNF-α and IL-1β significantly increased in APPsw-N2a cells treated with Mn, MCM, especially MCM from Mn-treated BV2 cells. APPsw-N2a cells were treated with a conditioned medium collected from BV2 cells in the absence or presence of 100 μM Mn for 24 h. *p < 0.05, **p < 0.01, ***p < 0.001, one-way ANOVA for **(A,B)**, two-way ANOVA for **(C)**, *n* = 5 for each group.

To examine the possibility that Mn enhancement of Aβ production/accumulation requires inflammatory cytokines from microglia, we analyzed the proinflammatory cytokines, IL-1β and TNF-α. Western blot and ELISA analyses showed that IL-1β and TNF-α levels were increased significantly in Mn-treated BV2 microglia culture supernatants compared with the control BV2 microglia cells (*n* = 5, *p* < 0.01, one-way ANOVA, Figures 7A,A1–A3). Then we used ELISA to measure the levels of IL-1β and TNF-α in the culture supernatants of APPsw-N2a cells treated with either Mn-containing or Mn-free MCM. We detected substantial differences in the level of IL-1β and TNF-α in the supernatants from the four groups of APPsw-N2a cell cultures: treatment with 100 μM Mn did not alter the level of IL-1β and TNF-α, treatment with MCM caused significant increase in the level of IL-1β and TNF-α (*n* = 5, *p* < 0.05, one-way ANOVA, Figures 7B,B1,B2); more important, treatment with 100 μM Mn-containing MCM further increased the levels of IL-1β and TNF-α (*n* = 5, *p* < 0.05, two-way ANOVA, Figures 7C,C1,C2).

**Figure 8:**
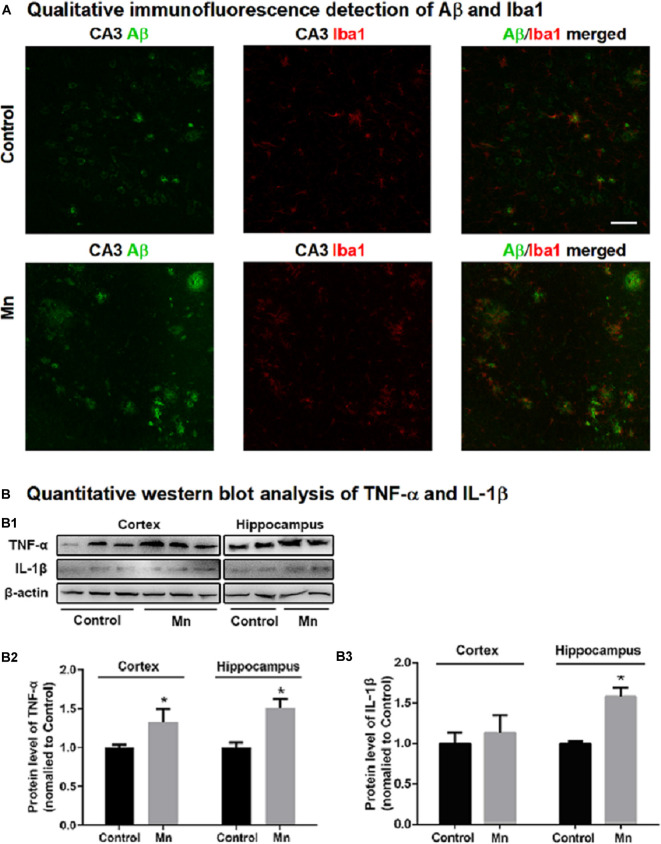
Mn treatment increased microglia inflammatory reaction in 3×Tg-AD mouse brains. **(A)** Qualitative exemplary confocal microscopic images showing that Aβ (green) was clearly increased, Iba1 signal (red, a marker for microglia) was stronger, and Ibal-labeled microglia were more numerous and in clusters, indicating microglial activation in the hippocampal CA3 area in chronic Mn-treated 3×Tg-AD mice. Scale bar = 40 μm. **(B: B1–B3)** Western blot quantitative analyses showing the expression levels of TNF-α and IL-1β in the cortex and hippocampus of the 3×Tg-AD mice following chronic Mn treatment. β-actin served as internal control. *p < 0.05, two-way ANOVA, *n* = 5 for each group.

To provide evidence for Mn activation of microglial cells in amyloidogenesis in intact animal AD models, we examined microglia in 3×Tg-AD mouse brain in the Mn group and Control group by immunostaining Iba1, a marker for microglia and its activation upon stimulation. As shown in Figure 8A, chronic Mn exposure increased Iba1-positive microglia and inflammatory foci in CA1, CA3, and DG of 3×Tg-AD mouse hippocampus compared with the Control group. Moreover, Iba1-positive microglia appeared in florid Aβ plaques in CA3 region of Mn-treated 3×Tg-AD mice (Figure 8A). In addition, Western blot analysis showed that chronic Mn exposure elevated the protein level of inflammatory cytokines IL-1β and TNF-α in the cerebral cortex and hippocampus (*p* < 0.05, two-way ANOVA; Figures 8B,B1–B3). These results provide evidence that in the 3×Tg-AD mice, chronic Mn exposure promote microglia activation and hence production and secretion of pro-inflammatory factors that in turn increase amyloidogenesis.

## Discussion

The main findings of our present study are that chronic increase in brain Mn enhances amyloidogenesis in 3×Tg-AD mice and cultured APPsw-expressing cells, and this Mn effect requires microglia activation and the likely release of inflammatory cytokines from activated microglia. These are novel findings that had not been reported before; thus our present study advances our understanding about Mn’s pro-amyloidogenic effects and the underlying cellular and molecular mechanisms.

### Chronic Increase in Brain Mn Increases APP Gene Expression and Amyloidogenic APP Protein Processing

While sufficient amounts of Mn are necessary for the human body and neurons to function normally (Horning et al., 2015), excessive Mn accumulation in the brain was shown to impair cognition and to be a risk factor for developing dementia and AD (Banta and Markesbery, 1977; Bowman et al., 2011; Tuschl et al., 2013; Tong et al., 2014; Wang et al., 2017), but the underlying molecular mechanisms are not fully understood. In young adult macaque monkeys, chronic (10 months) Mn overexposure upregulated the expression of amyloid-beta precursor-like protein-1, induced formation of diffuse Aβ plaques in the frontal cortex, and also triggered degenerative changes in cortical neurons of these Mn-treated monkeys (Guilarte et al., 2008; Guilarte, 2010). However, the molecular mechanisms of these Mn effects are not fully understood. Clearly, additional data are needed to more firmly establish Mn’s pro-amyloidogenic effects and the underlying molecular mechanisms.

Therefore, our present study examined the effects of Mn on the development of AD pathology in 3×Tg-AD mouse model (Oddo et al., 2003) and in a cell line stably expressing human Swedish mutant APP (APPsw-N2a cells). More important, because of the critical role of Aβ production in AD pathogenesis (Selkoe and Hardy, 2016; Forner et al., 2017; Walsh and Selkoe, 2020), we sought to identify the mechanisms by which Mn regulates Aβ generation and accumulation in the transgenic 3×Tg-AD mice (Oddo et al., 2003). We found that chronic Mn treatment increased Aβ plaques in the cerebral cortex and hippocampus, resulting from elevated APP gene and protein expression, BACE-1 gene and protein expression, and hence increased Aβ production and accumulation; simultaneously, Mn treatment reduced the non-amyloidogenic APP cleavage pathway by decreasing the expressing of ADAM10, a key component of α-secretase activity. These results indicate a new molecular basis for the clinical and experimental finding that high Mn in the brain is a risk factor to develop AD pathology and cognitive impairments, that is, Mn can increase both APP gene expression and amyloidogenic APP processing that produces Aβ 1–42. These are novel and important results that improve our understanding on Mn effects on AD pathogenesis.

We need to note here that in this study, the effects of Mn treatment were obtained by comparing baseline (no Mn-treated) 3×Tg-AD mice with Mn-treated 3×Tg-AD mice; such a comparison is a valid design that investigates if and how Mn affects Aβ production and accumulation in genetically AD-predisposed animals. Future studies will need to determine Mn’s potential amyloidogenic effects in normal animals.

### Mn Enhancement of Microglial Activation and Secretion of Inflammatory Cytokines Is Required for Mn Increase of Amyloidogenesis

Our results show, surprisingly, that Mn alone did not affect APP and BACE1 expression and Aβ generation; in APPsw-N2a cells, in contrast, when the APPsw-N2a cells were cocultured with microglia or cultured in a microglia-conditioned medium, Mn treatment increased the expression level of APP, BACE1, amyloidogenic C99 fragment, and Aβ; we also found that Mn treatment increased microglia-released inflammatory cytokines IL-1β and TNF-α in microglia culture and an increase in activated microglia in 3×Tg-AD mouse brains. These results are consistent with the fact that microglia are key innate immunoreactive cells in the brain that release proinflammatory cytokines when activated, and neuroinflammation is a key component of AD pathogenesis (Minter et al., 2016; Forner et al., 2017; Hansen et al., 2018; Nichols et al., 2019; Simon et al., 2019). It has been reported that Mn can induce microglia activation and neuroinflammation that caused hippocampal functional impairment (Wang et al., 2017). Previous studies also reported that Mn can activate microglia that in turn release proinflammatory cytokines IL-1β and TNF-α, leading to neuroinflammation, which in turn causes neuronal cell damage (Liu, 2006; Zhang et al., 2010; Park and Chun, 2017). Additionally, proinflammatory cytokines, especially IL-1β elevation has been detected in AD patients (Cacabelos et al., 1991; Griffin et al., 2000; Forlenza et al., 2009) and in the brains of aged AD model mice (Lim et al., 2000; Ghosh et al., 2013). Thus, our present findings are important. Our present study in 3×Tg-AD mice are also generally consistent with literature data indicating that Mn may impair hippocampus-dependent memory in rats and WT mice (Fu et al., 2016; Wang et al., 2017).

However, we need to note the following limitations of our results. First, besides IL-1β and TNF-α, microglia cells may release other factors that may partially mediate Mn-stimulated amyloidogenesis. Second, in our present study, we did not identify the states (M1 proinflammatory state vs. M2 state) of the microglial cells (Orihuela et al., 2016), but we speculate that the microglia cells associated with our observed effects were likely in the M1 state because we detected an increased release of IL-1β and TNF-α, and these proinflammatory cytokines that are commonly released from the M1 proinflammatory state. Future studies will need to determine these possibilities.

How Mn-triggered microglial IL-1β and TNF-α release subsequently increases APP production, BACE1 expression, and β-secretase activity (amyloidogenic) and decreases ADAM10/a-secretase activity (non-amyloidogenic) are currently not known, and even how Mn treatment affects IL-1β and TNF-α release was not settled in previous studies with conflicting results from cell and animal AD models (Domingues et al., 2017). Our present study has provided new data and advanced this field, although additional studies are required to obtain a more complete understanding.

## Concluding Remarks

**Figure 9:**
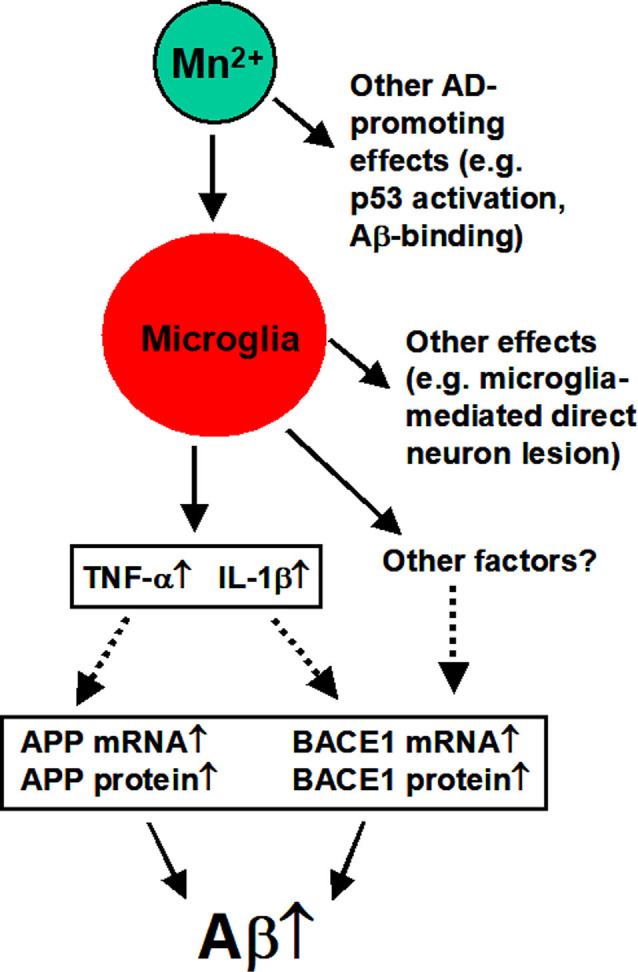
Diagram summarizing the key findings of our present study. Mn overexposure can activate microglia cells that release IL-1β and TNF-α that in turn increase APP expression and Aβ production and accumulation. Activated microglia cells may also produce other factors that may also promote amyloidogenesis (to be determined by future studies). Other effects are based on the literature as discussed in the text.

In summary, as diagramed in Figure 9, our study indicates that in the 3×Tg-AD mice and cultured APPsw-expressing cells, Mn increases amyloidogenic APP processing and Aβ production, and these Mn effects require microglia activation and the likely release of inflammatory cytokines. Thus, Mn enhancement of microglia activation cannot only lesion the brain neurons directly *via* the established cytokine-based mechanisms (Minter et al., 2016; Hansen et al., 2018; Nichols et al., 2019; Simon et al., 2019) but also by increasing APP expression and amyloidogenic APP processing, contributing to AD pathogenesis and neurodegeneration.

## Data Availability Statement

The raw data supporting the conclusions of this article will be made available by the authors, without undue reservation, to any qualified researcher.

## Ethics Statement

The animal study was reviewed and approved by the Laboratory Animal Ethics Committee of China Medical University which approved all experimental procedures.

## Author Contributions

GL, XL, and WZ: conceptulization, design, data collection and analysis, and manuscript writing. XC and NZ: data collection and analysis. All authors contributed to the article and approved the submitted version.

## Conflict of Interest

The authors declare that the research was conducted in the absence of any commercial or financial relationships that could be construed as a potential conflict of interest.
